# The subject-dependent, cumulative, and recency association of aerobic fitness with academic performance in Taiwanese junior high school students

**DOI:** 10.1186/s12887-018-1384-4

**Published:** 2019-01-17

**Authors:** Shu-Shih Hsieh, Jia-Ren Tsai, Shao-Hsi Chang, Jen-Yu Ho, Jui-Fu Chen, Po-Hsi Chen, Yao-Ting Sung, Tsung-Min Hung

**Affiliations:** 1 Department of Physical Education, No.162, Sec. 1, Heping E. Rd., Da’an Dist., Taipei, 10610 Taiwan, Republic of China; 2 Department of Statistics and Information Science, Fu Jen Catholic University, New Taipei, Taiwan; 3 Department of Athletic Performance, National Taiwan Normal University, Taipei, Taiwan; 4 Department of Educational Psychology and Counseling, National Taiwan Normal University, Taipei, Taiwan; 5 Institute for Research Excellence in Learning Science/Chinese Language and Technology Center, National Taiwan Normal University, No.162, Sec. 1, Heping E. Rd., Da’an Dist., Taipei, 10610 Taiwan, Republic of China

**Keywords:** Fitness, Language, Math, Science, Social science, Adolescence

## Abstract

**Background:**

The objective of the current study was to examine whether the relation between aerobic fitness and academic achievement during adolescence is subject-dependent, and to investigate *cumulative* and *recency* effects.

**Methods:**

This study made use of two nationwide datasets. The first was the aerobic fitness profile of junior high school students collected by the Ministry of Education in Taiwan. The second contained the scores on the Basic Competence Test for Junior High School Students (BCTJH). The sample consisted of 382,259 students who completed the BCTJH in the 5 years between 2009 and 2013. Data on each student’s aerobic fitness during their three years of junior high school were matched with their exam results at the end of this period.

**Results:**

The results revealed that **s**tudents classified as highly-fit during at least one of the three years had higher BCTJH scores than those who never achieved this level, with the size of effect increasing with the length of time that fitness was maintained. Additionally, aerobic fitness in the final year was more closely linked to BCTJH scores than that in the earlier two years. Fitness was also more strongly associated with exam performance in math, science and social science, relative to language-related subjects.

**Conclusions:**

Our findings suggest that while aerobic fitness is positively related to academic achievement in Taiwanese junior high school students, the relationship depends on academic subject, as well as the length and time of being aerobically fit.

**Electronic supplementary material:**

The online version of this article (10.1186/s12887-018-1384-4) contains supplementary material, which is available to authorized users.

## Background

Research has shown a close relation between higher physical fitness and superior academic achievement in adolescents. This relation has been supported by cross-sectional (1, 2), longitudinal (2), and interventional studies (2). Cross-sectional studies have found aerobic fitness to have the strongest relation to scholastic performance relative to other fitness domains [1, 2] and this relation was recently also demonstrated in the case of fitness over 3 years [3–5]. This association could be accounted for by neurobiological changes induced by higher aerobic fitness, such as increased cerebral blood flow in brain areas associating with learning (e.g., hippocampus) [6], better in-task functioning [7], stronger white matter integrity [8], better ability to stay focused [9], and better language [10] or arithmetic processing [11]. It is therefore reasonable to expect that aerobic fitness would translate into greater academic achievement.

It would be useful to examine whether the relation between aerobic fitness and academic achievement in adolescents differs by academic subject. Cross-sectional studies [12–14] have reported a stronger relation of aerobic fitness with math-related subjects relative to language-related ones. Data from longitudinal studies, however, have been inconsistent. For example, whereas Raine et al. [15] found that changes in aerobic fitness over 3 years was more strongly linked to math relative to reading performance, Sardinha et al. [16] found that adolescents who maintained high levels of aerobic fitness over 3 years were more likely to obtain high marks in language and foreign language subjects than math or science. The reason for this inconsistency may relate to the fact that while Sardinha et al. used academic metrics based on a combination of written exams and class participation, whereas the study by Raine et al. was primarily based on written exams. Besides there being differences in the reliability of these two measures, neither of the studies looked at a wide range of academic subject measures.

Previous studies [3, 4, 16] have looked at fitness levels at the beginning and end of a 3-year period but did not investigate fitness in the interim. If aerobic fitness is truly related to academic performance, it is reasonable to assume that a “*cumulative*” level of fitness across the 3-year period would determine final academic achievement. Students who maintain a high level of fitness throughout a 3-year period should perform better on exams than those who were only fit in two of the three years, and they in turn should outperform those who were only fit in a single year. This hypothesis is supported, at least in part, by Hillman et al. [17] and Krafft et al. [8] who found that students with a higher level of attendance at aerobic fitness classes, and who might then be assumed to have had higher aerobic fitness, showed relatively better cognitive function [15] and increased white matter integrity [8]. It is also possible that fitness levels in year 3 would have a disproportionately stronger effect on academic performance than that in earlier years because student’s fitness may coincide with a time where material more likely to appear on the exam are being taught (i.e. there may be a “*recency*” effect). To the best of our knowledge, these issues have not been previously investigated.

In summary, the questions addressed by the current study were three-fold: (a) whether higher cumulative levels of aerobic fitness associated with better exam performance at the end of the period; (b) whether aerobic fitness in the third year (i.e., closer in time to when the measure of academic achievement occurred) more strongly associated with academic performance than earlier years; and (c) whether the associations between fitness and academic achievements vary by academic subjects. To investigate the importance of *cumulative* and *recency* effects of aerobic fitness, we analyzed fitness data from all three of the years of junior high school in Taiwan. It was hypothesized that a higher cumulative level of aerobic fitness would be associated with better exam performance, while aerobic fitness in the third year would have a stronger association with academic achievement relative to earlier years. However, due to the lack of consistency of prior studies, no predictions were made in relation to whether the relation between fitness and academic achievement would differ between academic subjects.

## Methods

### Design and participants

The current study examined the ability of changes over a 3-year period in aerobic fitness to predict academic performance at the end of the period. Data on a total of 398,870 junior high school students between the ages of 12 to 15 (Mean_year 1_ = 12.8, SD_year 1_ = 0.5; Mean_year 2_ = 13.8, SD_year 2_ = 0.5_;_ Mean_year 3_ = 14.8, SD_year 3_ = 0.5) were collected from 5 cohorts of students attending junior high school in Taiwan during 2006–2009, 2007–2010, 2008–2011, 2009–2012, and 2010–2013. The retrieval of data from students was conducted in October 2014 and was approved by the ethics review panel of the Ministry of Education in Taiwan (MOE). No informed consent form from students was required from the ethics review panel because we simply de-identified students’ data. All data, including students’ fitness and academic data, were retrived anonymously. Details of data usage and matching is explained below.

### Data and settings

Data collection took place from September of 2006 to May of 2013. The current study made use of two datasets. The first dataset contained a physical fitness profile of junior high school students collected by the MOE. Every junior high school student in Taiwan was required to take these tests within the first 4 weeks of each academic year (which starts in September) over the 3-year period. The second dataset contained scores on the Basic Competence Test for Junior High School Students (BCTJH), a compulsory examination administered by the MOE at the end of junior high school (at the end of May) to all high-school-bound students.

Students’ physical fitness and BCTJH scores were matched on their name and personal identification number. Data from high-school-bound juniors who took the BCTJH in 2009, 2010, 2011, 2012, and 2013 in their third year were examined, with fitness scores obtained at the beginning of the first and third year being matched with BCTJH scores at the end of the third year. For example, students who sat the BCTJH in May of 2013 had their test results matched with their fitness scores from September, 2010 and September, 2012. Analogous procedures were applied to students who sat for the BCTJH in the other four years (i.e., 2009, 2010, 2011, and 2012).

### Measurements

Aerobic fitness was assessed by a 1600- and 800-m run test for boys and girls at the beginning of each academic year. This test is a standard measure of aerobic fitness used for Taiwanese adolescents [3, 4, 18], and has been shown to have a high criterion-related validity (*r* = 0.79) [19]. Students were instructed to give their best effort to run/walk the distance as fast as possible. The score on this test was the total time in seconds to cover the 1600- or 800-m distance, with shorter time indicating better performance.

Academic achievement was assessed by the BCTJH at the end of the final year. The BCTJH is a compulsory, nationwide examination given to all high-school-bound students in Taiwan. This measure is not only a standardized and validated measure of academic achievement, students are also highly motivated to do their best when taking the exam since the results determine their chances of being admitted to competitive high schools which, in turn, will affect their future educational and career path [20, 21]. This test consists of 6 subjects: language (Chinese), foreign language (English), math, social science, science, and an essay. The first five tests consist of computer-scored, multiple choice questions. Scale scores ranging from 1 to 80 points are determined for each test based on performance on the questions answer correctly. The essay, which was marked by trained MOE examiners, had a maximum score of 12 and required students to write down their thoughts on a given topic. Thus, the maximum obtainable score on the BCTJH was 412 points. The current study extracted data from all subjects except the essay due to its subjective nature and low discriminatory validity (only 6 scores were given, 2, 4, 6, 8, 10, and 12, with the vast majority of students being given a 6 or 8). In practice, even very small differences in exam performance often have significant implications. For example, a 2 to 4 points increase in total score might typically allow entry to a high school standing one or two places higher in the national rankings.

In addition to aerobic fitness and academic performance, data were collected on sex and degree of urbanization (classified into 3 groups of high, medium and low urbanization) of the region where students’ schools were located. Urbanisation was assessed on the following parameters: 1) population density; 2) average educational level; 3) percentage of citizens over 65 years old); 4) percentage of the population engaged in agricultural work; and 5) ratio of the number of physicians to the total population. In addition, body mass index (BMI) data was obtained at the same time as aerobic fitness (i.e., at the beginning of each academic year) by measuring height and weight as a surrogate measure of body composition. BMIs of students were classified as thin, normal, overweight, or obese based upon age- and sex-adjusted norms provided by the MOE (Additional file 1: Supplement).

### Data analysis

Levels of aerobic fitness in the first, second, and third years (corresponding to the seventh, eight, and ninth grades) were classified as “*Highly-fit*” if their scores were in the top 25% in that year as determined by age- and sex-adjusted MOE norms, and classified as “*Not highly-fit*” otherwise. This study used the top 25% as the cut-off criteria, which has been adopted elsewhere [4], to truly reflect the idea of being ‘*fit*’. Five groups were identified based on fitness patterns over the three years of junior high school to address the ‘*cumulative*’ and ‘*recency*’ effects of aerobic fitness: (1) highly fit in all years (3F); (2) *highly fit* in *any* two of the three years (e.g., year 1 and 3, year 1 and 2, or year 2 and 3) (2F); (3) highly fit only in year 3 (F3 group); (4) highly fit in either year 1 or year 2 but not both (1F group), and (5) not highly fit in any of the three years (0F group). This group comparison design was well suited to determining how students with different fitness patterns differed from each other in term of exam scores and to gauge the practical significance of this effect.

Data were analyzed using SPSS 21.0, with an alpha of 0.05 set as the threshold for statistical significance. Pearson product-moment correlation coefficients were first computed to see whether demographic variables (i.e., sex, BMI, level of urbanization) were correlated with fitness patterns and/or exam scores. All data were converted to dummy variables with the exceptions of scores on the five academic subjects. Next, one-way analyses of covariance (ANCOVAs) were separately performed with fitness pattern group as the between-subjects factor. The scores of individual subjects were as the dependent variables. Sex, BMI, and degree of urbanization were used as covariates if found to be correlated with either fitness patterns or exam scores. *Bonferroni*-corrected *t*-tests were utilized for post hoc analyses. For interpretative purposes, Cohen’s *d* effect sizes and % differences in test scores were calculated when necessary. The following conventions were used to determine the magnitude of the *d* effect sizes: 0.2, 0.5, 0.8 to represent small, medium, and large effect sizes, respectively [22].

To more clearly address the subject-dependent nature of the fitness-achievement relationship, a comparison was made between the average exam scores of those who were judged to be highly fit at any time during the 3-year period (i.e., (3F + 2F + F3 + 1F)/4)) with the 0F group for each academic subject.

## Results

Results of the bivariate correlations showed that all demographic variables, including sex, BMI type, and level of urbanization were correlated with fitness patterns (*r*’s = −.059–.180, *p*’s < .001) as well as scores in language (*r*’s = −.068–.032, *p*’s < .001), foreign language (*r*’s = −.112–.039, *p*’s < .001), math (*r*’s = −.052–.046, *p*’s < .001), science (*r*’s = −.054–.066, *p*’s < .001), and social science (*r*’s = −.050–.033, *p*’s < .001). Thus, all these demographic variables were included as covariates in the subsequent analyses.

Table 1 shows test scores as a function of group. Regarding language, the effect of *Group* was significant, *F*(4, 382,251) = 407.38, *p* < 0.001, η_p2_ = 0.004. Pairwise comparisons showed that the 3F, 2F, F3, and 1F groups had higher scores than the 0F group. Scores of the 3F group were higher than that in the 2F, F3, or 1F groups. Scores in the 2F and F3 groups were higher than that in the 1F group.

**Table 1:** Descriptive data of test scores within subject groups

Groups	M (SD)	Cohen’s *d* and % difference compared to 0F group
Language		
3F group (*n* = 37,363)	55.3 ± 19.1	0.20; 7.6%
2F group (*n* = 46,278)	53.7 ± 19.2	0.12; 4.5%
F3 group (*n* = 24,868)	53.3 ± 19.2	0.10; 3.7%
1F group (*n* = 51,203)	52.2 ± 19.2	0.04; 1.6%
0F group (*n* = 222,547)	51.4 ± 19.2	–
Foreign language		
3F group (*n* = 37,363)	56.3 ± 23.4	0.21; 9.5%
2F group (*n* = 46,278)	54.3 ± 23.7	0.12; 5.6%
F3 group (*n* = 24,868)	53.7 ± 23.6	0.10; 4.5%
1F group (*n* = 51,203)	52.6 ± 23.7	0.05; 2.3%
0F group (222547)	51.4 ± 23.7	–
Math		
3F group (*n* = 37,363)	56.5 ± 19.5	0.29; 11.2%
2F group (*n* = 46,278)	54.4 ± 19.7	0.18; 7.1%
F3 group (*n* = 24,868)	53.7 ± 19.5	0.15; 5.7%
1F group (*n* = 51,203)	52.3 ± 19.8	0.08; 3.0%
0F group (222547)	50.8 ± 19.8	–
Social Science		
3F group (*n* = 37,363)	56.3 ± 20.9	0.24; 10.0%
2F group (*n* = 46,278)	54.3 ± 21.2	0.15; 6.1%
F3 group (*n* = 24,868)	54.0 ± 21.0	0.13; 5.5%
1F group (*n* = 51,203)	52.4 ± 21.2	0.06; 2.3%
0F group (222547)	51.2 ± 21.0	–
Science		
3F group (*n* = 37,363)	56.3 ± 18.8	0.30; 10.8%
2F group (*n* = 46,278)	54.3 ± 18.8	0.19; 6.9%
F3 group (n = 24,868)	53.7 ± 18.7	0.16; 5.7%
1F group (*n* = 51,203)	52.2 ± 18.7	0.08; 2.8%
0F group (*n* = 222,547)	50.8 ± 18.5	–

The effect of *Group* was significant on foreign language, *F*(4, 382,251) = 425.37, *p* < 0.001, η_p2_ = 0.004. Post hoc comparisons showed that the 3F, 2F, F3, and 1F groups had higher scores than the 0F group. Scores for those in the 3F group were higher than those in the 2F, F3, and 1F groups. Scores in 2F and F3 groups were higher than that in the 1F group.

With respect to math, the effect of *Group* was significant, *F*(4, 382,251) = 721.50, *p* < 0.001, η_p2_ = 0.007. Pairwise comparisons showed that the 3F, 2F, F3, and 1F groups had higher scores than the 0F group. Scores for the 3F group were higher than that for those in the 2F, F3, and 1F groups. Scores for the 2F group were higher than the F3 and 1F groups. The F3 group scored higher than the 1F group.

Analysis on social science results found a main effect of *Group*, *F*(4, 382,251) = 513.70, *p* < 0.001, η_p2_ = 0.005. Post hoc analyses showed that the 3F, 2F, F3, and 1F groups had higher scores than the 0F group. The 3F group scored higher than those in the 2F, F3, and 1F groups. Scores in the 2F and F3 groups were higher than the 1F group.

Analysis on science data found a main effect of *Group*, *F*(4, 382,251) = 749.36, *p* < 0.001, η_p2_ = 0.008. Post hoc analyses showed that the 3F, 2F, F3, and 1F groups had higher scores than the 0F group. The scores of those in the 3F group were higher than those in the 2F, F3, and 1F. The 2F group scored higher than the F3 and 1F groups and the F3 group scored higher than that in 1F group.

Thus, the results for all five academic subjects provided support for the existence of both *cumulative* (i.e., 3F > 2F > 1F) and *recency* effects (i.e., F3 > 1F).

Table 2 shows a comparison between the average scores across the 3F, 2F, F3, and 1F groups against the 0F group. It is evident that the degree of outperformance of the former depended on the examination subject. Specifically, in comparison to students who were never highly-fit, students who were highly fit for at least one year showed an average improvement in test scores of 2.2 points (4.3%) in language, 2.8 points (5.5%) in foreign language, 3.1 points (6.1%) in social science, 3.3 points (6.5%) in science, and 3.4 points (6.7%) in math. These data suggest a subject-dependent association.

**Table 2:** Summary of the relationship between aerobic fitness and academic performance in various subjects

	Average scores of 3F, 2F, F3, and 1F groups	0F group	% of outperformance relative to 0F group
Math	54.2	50.8	6.7%
Science	54.1	50.8	6.5%
Social science	54.3	51.2	6.1%
Foreign language	54.2	51.4	5.5%
Language	53.6	51.4	4.3%

*Note.* 3F group = highly fit in all years; 2F group = highly fit in any two years; F3 group = highly fit only in year 3; 1F group = highly fit in year 1 or 2 but not both; 0F group = never highly fit

## Discussion

The main findings of the current study are that: (a) aerobic fitness was more strongly associated with performance in math- and science-related subjects relative to language-related ones; (b) the longer students maintained a high level of aerobic fitness, the better their academic performance, with those who maintained a high level of aerobic fitness throughout junior high school having the strongest academic performance, followed by those who were highly fit in two of the three years, and then by those with a high level of fitness in only one of the three years; and (c) among those who were only highly fit in one of the three years, being fit in year 3 (i.e., the F3 group) had a stronger effect on academic performance than being fit in either year 1 or 2 (i.e., the 1F group). In the case of performance in language, foreign language, and social science, those who were only fit in year 3 were not significantly different from those who were fit for two years.

Previously, cross-sectional [12–14] and longitudinal studies [15] have reported that while aerobic fitness is closely related to performance in language and math-related tests, the relation is stronger in the case of the latter. The findings of the current study have supported these results showing a positive, long-term relation between aerobic fitness and academic performance [3–5, 15, 16, 23, 24] using a comprehensive and standardized measure of academic achievement, with the association being stronger in math, science, and social science relative to languge and foreign language. This finding may imply that the strength of association between aerobic fitness and academic performance depends on the degree of reliance on high-order cognitive control (e.g., analytical thinking, self-monitoring, reasoning, flexibility, working memory) required for a given subject. This speculation is supported by the fact that neuropsychological findings have shown a strong association between higher aerobic fitness and better cognitive control [25, 26] and relatively weak association with verbal fluency or stimuli recall [27].

Another contribution of the current study is its finding that the longer high fitness levels were maintained, the better the exam results. On average, students who were classified as highly fit in all three years outperformed those classified as highly fit for 2 years and 1 year by 2 and 4 points, respectively. These differences in exam scores are sufficiently large to make a practical difference in the quality of high school to which students would be allowed to attend. This existence of a *cumulative* effect has been reported by Hillman et al. [17] and Krafft et al. [8] who found that students with a higher level of attendance at aerobic fitness classes, who can therefore be assumed to have had higher levels of aerobic fitness, showed relatively better performance in executive cognition [17] and increased white matter integrity [8]. Our findings have provided additional support for the close relation between cumulative aerobic fitness and academic performance previously indicated by cross-sectional studies during childhood and adolescence [28–30], and have extended previous studies that reported fitness data from only year 1 and year 3 of junior high school [3, 4, 16].

A *recency* effect was also found, with students with a high level of fitness only in year 3 outperformed those who were only highly fit in year 1 or year 2. The level of outperformance (i.e., one or two points) is sufficient to have a meaningful impact on high school opportunities. In fact, in the case of language, foreign language, and social science, the exam performance of students who were only highly fit in year 3 was not inferior to those who were highly fit in two of the three years. In addition to the possibility that student’s fitness, and therefore greater learning ability, coincided with a time where material more likely to appear on the exam are being taught. This might have meant that they performed better due to an enhanced ability to stay focused [9], a richer network of words and meanings [10], and a improved strategic selection ability.

There are several limitations to the current study. Firstly, the experimental design employed does not allow a definitive conclusion to be made as to whether the relation between fitness and examination performance was causal in nature. Nevertheless, it is likely that this is the case given that: (a) the fitness measures were taken some considerable time before sitting the BCTJH; and (b) a ‘dose-related’ relation between aerobic fitness and academic performance was observed, suggesting that exam performance improved in proportion to the time being aerobically fit.

Secondly, the use of junior high school students as experimental subjects means that the results reported here may not be generalizable to other school aged populations given the possible effects of developmental factors on cognitive domains responsible for learning (e.g., working memory) [31].

Thirdly, given that a dichotomized measure of fitness was used, it remains unclear whether the relation between levels of aerobic fitness and academic achievement is linear. Previous study has suggested that the relation may, in fact, be nonlinear [13]. The use of the top 25% cut-off point may omit information regarding the relation between fitness and academic performance in individuals with general fitness. Future study is therefore strongly recommended investigatinge the relation using multiple levels of fitness categorization.

Fourthly, no measure of socioeconomic status (SES) was taken and thus it was not possible to directly control for this variable. Nevertheless, the inclusion of ‘level of urbanization’ as a covariate may have gone some ways toward achieving this, since its definition included several variables (e.g. educational level, percentage of the population engaged in agricultural work) which are strongly linked to SES.

Likewise, there were numbers of factors relating to fitness and/or academic performance, such as cognitive function [32], intelligent ability [33], nutrition [34], after-curriculum physical activity [34], presence of neuropsychological disorders or special education needs (e.g., learning difficulties) [35, 36], and time in transportation by motorized vehicle [37] that were not considered by the current study and most of the previous studies. It is recommended that future research investigate the effects of these factors further.

Lastly, the fitness norms established by the MOE were based on performance within the Taiwanese population and may not be comparable to other fitness norms, such as those published by the American College of Sports Medicine [38]. Nevertheless, the difference between norms should be relatively small since it is known that the physical fitness of Taiwanese adolescents is comparable to adolescents from other countries, as least those in the Asian region [39].

## Conclusions

The current study is the first to examine the relation between aerobic fitness across the three years of junior high school with different measures of academic achievement. The major findings of this study are that: (a) the relation between aerobic fitness and academic achievement depends on academic subject, with fitness being particularly beneficial for subjects having stronger reliance on executive cognition, such as math and science-related subjects, relative to language-related ones; and (b) while maintaining a high level of aerobic fitness throughout the 3 year period produced the greatest exam benefits, there was disproportional effects from being fit in the final year of school.

Recently, many schools are increasing instructional time in academic subjects such as math, reading, and science in an attempt to improve academic performance. However, many non-academic activities such as physical education are being cut from the school day [40]. This is also the case in Taiwan, where the requirement for physical education classes in junior high school (i.e., 90 min per week) is far less than the 60-min (or more) daily physical exercise requirement suggested in the guidelines of the American College of Sports Medicine [38]. Considering the positive relations between aerobic fitness with different academic subjects, educators and/or policy makers might consider increasing time allocated to aerobic exercise in the school curriculum as part of a strategy to enhance academic performance.

## Additional file

Additional file 1: Supplementary file Age- and sex-adjusted norms of BMI in Taiwanese students. (DOC 37 kb)

## Abbreviations

BCTJH: Basic Competence Test for Junior High School Students
MOE: Ministry of Education in Taiwan
