# Supplementary material for: The subject-dependent, cumulative, and recency association of aerobic fitness with academic performance in Taiwanese junior high school students
Source: BMC Pediatr. 2019 Jan 17;19:25. doi: 10.1186/s12887-018-1384-4 (PMC6337796; doi:10.1186/s12887-018-1384-4)
Supplement: Supplementary file 1 — Supplementary file Age- and sex-adjusted norms of BMI in Taiwanese students. (DOC 37 kb) [file 12887_2018_1384_MOESM1_ESM.doc]

**Supplement**

Boys

| Age | Thin | Normal | Overweight | Obese |
| --- | --- | --- | --- | --- |
| 12 | ≦15.1 | 15.2 | 21.3 | ≧23.9 |
| 13 | ≦15.6 | 15.7 | 21.9 | ≧24.5 |
| 14 | ≦16.2 | 16.3 | 22.5 | ≧25.0 |
| 15 | ≦16.8 | 16.9 | 22.9 | ≧25.4 |

Girls

| Age | Thin | Normal | Overweight | Obese |
| --- | --- | --- | --- | --- |
| 12 | ≦15.1 | 15.2 | 21.3 | ≧23.9 |
| 13 | ≦15.6 | 15.7 | 21.9 | ≧24.3 |
| 14 | ≦16.2 | 16.3 | 22.5 | ≧24.9 |
| 15 | ≦16.6 | 16.7 | 22.9 | ≧25.2 |
